# Delayed epiphyseal closure in an adult with panhypopituitarism detected by ^99m^Tc‐MDP bone SPECT/CT

**DOI:** 10.1002/ccr3.7644

**Published:** 2023-07-04

**Authors:** Yu Ning, Xiaoji Liu, Yao Chen, Min Cai, Sijin Li

**Affiliations:** ^1^ Department of Medical Imaging Shanxi Medical University Taiyuan China; ^2^ School of Forensic Medicine Shanxi Medical University Taiyuan China; ^3^ Department of Nuclear Medicine Shanxi Provincial People's Hospital & Fifth Hospital of Shanxi Medical University Taiyuan China; ^4^ Department of Nuclear Medicine First Hospital of Shanxi Medical University Taiyuan China

**Keywords:** ^99m^Tc‐MDP, delayed epiphyseal closure, panhypopituitarism, SPECT/CT

## Abstract

We reported a 23‐year‐old male patient with panhypopituitarism who underwent two resections for craniopharyngioma and received postoperative hormone replacement therapy. The ^99m^Tc‐MDP bone scan revealed focal high uptake of radioactive nuclide in multiple large joints. The SPECT/CT demonstrated the focal high uptake in their metaphysis. Thus, delayed epiphyseal closure was considered.

## Graphical abstract

^99m^Tc‐MDP wholebody bone scan and SPECT/CT of the knee joint.

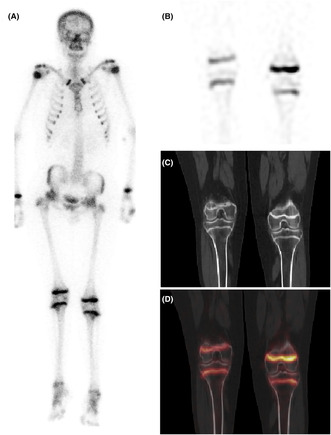

## 1 BACKGROUND

Panhypopituitarism was defined as the syndrome of insufficient secretion of one or more pituitary hormones caused by partial or complete loss of pituitary function, with clinical features including short stature, reduced growth velocity and bone mineral density, delayed development of secondary sexual characteristics, delayed epiphyseal closure (where the long bones continue to grow), tiredness, cold intolerance, weight gain, constipation, and dry skin.^1^ Normally, the average age for epiphyseal closure of the knee and ankle joints in male adolescents is 17 years of age.^2^ Factors affecting the epiphysis closure mainly include hormone levels, nutritional status, and geographical factors.^3^

The delayed epiphyseal closure in this case was believed to be the consequence due to hypohypophysis after craniopharyngioma resections, which resulting in insufficient secretion of pituitary hormones,^4^ such as sex hormones, growth hormone, and thyroid hormone.

## 2 CASE REPORT

**FIGURE 1:**
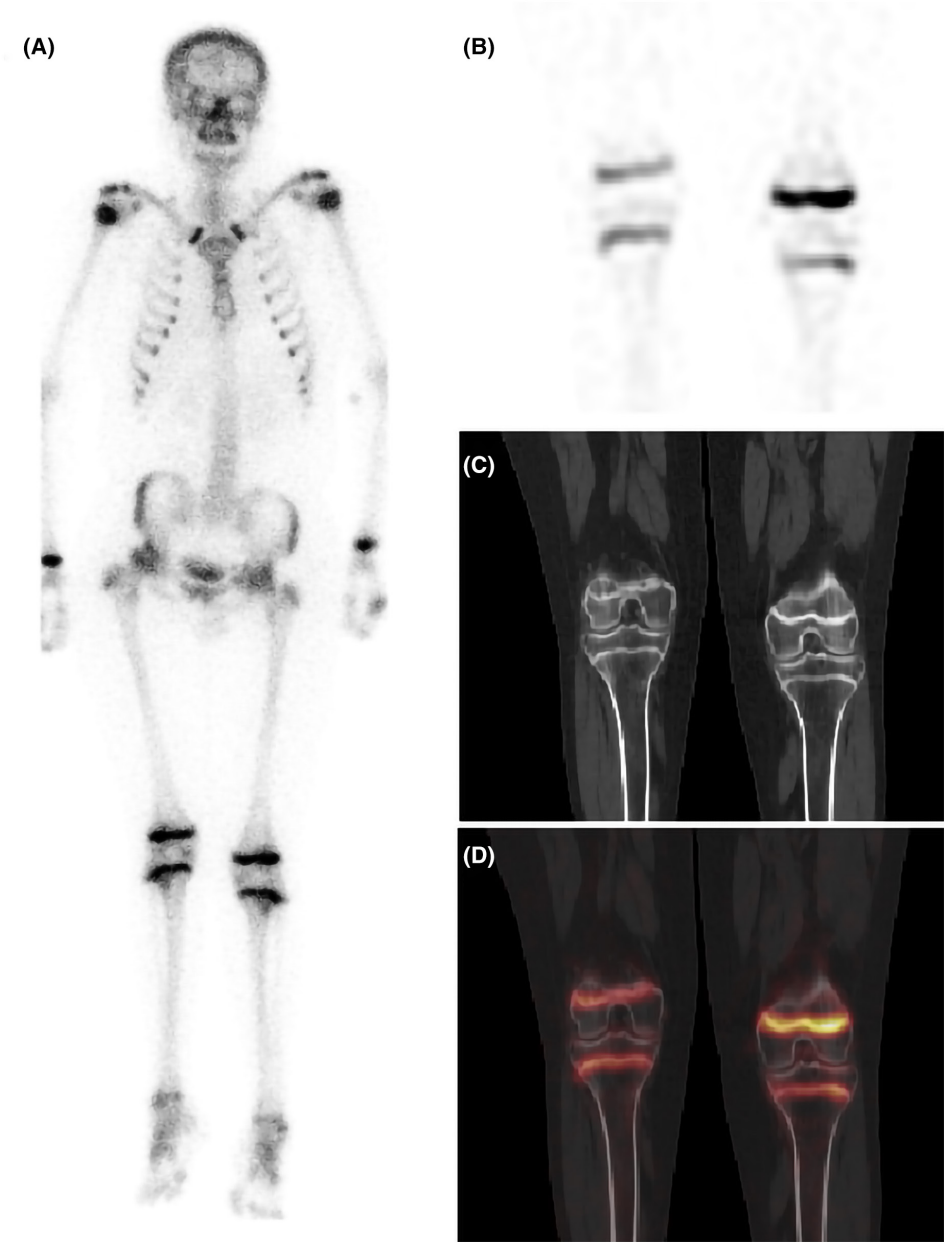
(A) Whole‐body bone scan showing intense uniform symmetric tracer uptake in long bone epiphyses such as bilateral humeral heads, wrist joints, knee joints, and ankle joints. (B) Local SPECT of the knee joints showing the radiotracer concentrates in epiphyses of bilateral lower femurs and upper tibias. (C) On CT images, the epiphyses are not closed in the bilateral knee joints, and the epiphyseal lines are symmetrically thickened, with density increased. (D) SPECT/CT fusion image confirms the increased radioactivity is located in the epiphyseal lines.

A 23‐year‐old male patient underwent two craniopharyngioma surgeries, one 12 years ago and another 2 years ago. He has been complaining of pain in both lower extremities for the past years. Since his first surgery, he has been regularly taking hydrocortisone acetate, thyroid hormone, and antiepileptic medications, but his sex hormones' taking was irregularly. Physical examination revealed dysplastic secondary sexual characteristics, and his testes and penis were at Tanner stage І. Laboratory tests (Table 1) showed that his serum sex hormones, thyroid hormone, and insulin‐like growth factor‐1 (IGF‐1) were lower than normal values though his growth hormone was within the normal range. He was diagnosed “panhypopituitarism” and hospitalized. The radiograph displayed bilateral femoral head necrosis. To rule out other possible skeletal diseases, he was scheduled for a whole‐body bone scan (Siemens Symbia Intevo, Germany). It was performed 2 h after intravenous administration of 25.0 mCi (925 MBq) ^99m^Tc‐methylene diphosphonate (^99m^Tc‐MDP). Intense, uniform, and symmetric tracer uptake was seen in multiple long bone epiphyses, such as bilateral humeral heads, wrist joints, knee joints, and ankle joints, which were rare in adults, suggesting active bone growth (Figure 1A). To further confirm the diagnosis, local SPECT/CT was performed on the knees. In SPECT (Figure 1B), the radiotracer concentrated in the epiphysis of bilateral lower femurs and upper tibias. CT image (Figure 1C) displayed that the epiphysis was not closed, and the epiphysis lines were symmetrically thickened, with increased density. SPECT/CT fusion images (Figure 1D) confirmed the increased radioactivity was localized in the epiphyseal lines. As a result, this patient's condition was considered delayed epiphyseal closure.

**TABLE 1:** Laboratory values.

Laboratory parameter	Value	Reference range
IGF‐1	<2.5 ng/mL	Adults over 20 years old: 60–350 ng/mL
T3	1.01 nmol/L	1.3–2.6 nmol/L
T4	48.4 nmol/L	58–161 nmol/L
TSH	8.57 nmol/L	0.4–4.0 nmol/L
LH	<0.1 mIU/mL	Male: 1.1–25.0 mIU/mL
TEST	1.696 ng/mL	Male: 2.2–10.5 ng/mL
FSH	1.06 mIU/mL	1.27–19.26 mIU/mL
hGH	0.032 ng/mL	0.003–0.971 ng/mL

Abbreviations: FSH, follicle‐stimulating hormone; hGH, human growth hormone; IGF‐1, insulin‐like growth factor‐1; LH, luteinizing hormone; T3, triiodothyronine; T4, thyroxine; TEST, testosterone; TSH, thyroid‐stimulating hormone.

## 3 DISCUSSION

Epiphyseal cartilage is a piece of cartilage that is present in the metaphysis of adolescents, promoting bone growth through the continuous division, proliferation, and ossification of its internal chondrocytes. Epiphyseal fusion occurs after the depletion of chondrocytes' proliferation.^5^ In this case, panhypopituitarism after craniopharyngioma surgeries resulted in pituitary hormone insufficiency,^6^, ^7^ such as luteinizing hormone (LH). LH stimulates Leydig cells to secret testosterone, which then converts to dihydrotestosterone and estradiol.^8^ Testosterone promotes secondary sexual characteristics in men. Estradiol, the most biologically active hormone in estrogen, promotes the development of secondary sexual characteristics in women and plays an important role in epiphyseal closure in both males and females. It acts directly in epiphyseal plates to accelerate their senescence, and promote the closure of the epiphysis.^9^, ^10^, ^11^, ^12^, ^13^ This male patient's epiphyses remain active due to deficient estrogen. At the same time, his long bones keep growing because the growth hormone is at normal level. This patient needs further treatment with sex hormones.

The ^99m^Tc‐MDP bone scan is a sensitive detection method for osteoblastic activity.^14^ Bone SPECT/CT could quantify osteoblast activity in the epiphyseal plate.^15^ There is a close correlation between the bone growth rate and the standard uptake value (SUV) of ^99m^Tc‐MDP.^15^ In normal children and adolescents, the uptake of ^99m^Tc‐MDP is increased in metaphyseal areas of extremities. This status usually disappears at the age of 18 to 20. Increased ^99m^Tc‐MDP uptake in adult epiphysis often indicates delayed epiphyseal closure. Additionally, abnormal uptakes of ^99m^Tc‐MDP and ^18^F‐fluorodeoxyglucose (^18^F‐FDG) in epiphyses could also reveal other bone diseases, such as post‐transplant distal limb syndrome,^16^ hypophosphatemic rickets,^17^ and primary bone lymphoma.^18^

## 4 CONCLUSION

Osteoblast metabolism is vigorous in the epiphyseal cartilages of delayed closure, and a large amount of bone imaging agents can be taken up. ^99m^Tc‐MDP accumulates in the sites of increased osteoblast activity, appearing as radioactive concentration. Therefore, ^99m^Tc‐MDP bone SPECT/CT could be used to detect delayed epiphyseal closure in adults and could help evaluate the therapeutic effects.

## AUTHOR CONTRIBUTIONS

**Yu Ning:** Data curation; investigation; writing – original draft. **Xiaoji Liu:** Data curation; investigation; writing – original draft. **Yao Chen:** Data curation; methodology; writing – original draft. **Min Cai:** Funding acquisition; writing – review and editing. **Sijin Li:** Writing – review and editing.

## FUNDING INFORMATION

The study was supported by the China Postdoctoral Science Foundation (No. 2020M670704) and the Fundamental Research Program of Shanxi Province (No. 202103021224377).

## CONFLICT OF INTEREST STATEMENT

The authors declare that there is no conflict of interest.

## ETHICS STATEMENT

The article describes a case report. Therefore, no additional permission from our Ethics Committee was required.

## CONSENT

Written informed consent was obtained from the patient to publish this report in accordance with the journal's patient consent policy.

## Data Availability

Data sharing is not applicable to this article as no new date were created or analyzed in this study.
